# Longitudinal Plasma Proteomics-Derived Biomarkers Predict Response to MET Inhibitors for MET-Dysregulated NSCLC

**DOI:** 10.3390/cancers15010302

**Published:** 2023-01-01

**Authors:** Guang-Ling Jie, Lun-Xi Peng, Mei-Mei Zheng, Hao Sun, Song-Rong Wang, Si-Yang Maggie Liu, Kai Yin, Zhi-Hong Chen, Hong-Xia Tian, Jin-Ji Yang, Xu-Chao Zhang, Hai-Yan Tu, Qing Zhou, Catherine C. L. Wong, Yi-Long Wu

**Affiliations:** 1 School of Medicine, South China University of Technology, Guangzhou 510006, China; 2 Guangdong Lung Cancer Institute, Guangdong Provincial People’s Hospital (Guangdong Academy of Medical Sciences), Southern Medical University, Guangzhou 510080, China; 3 Department of Clinical Skills Training Center, Zhujiang Hospital, Southern Medical University, Guangzhou 510282, China; 4 Department of Hematology, First Affiliated Hospital, Jinan University, Guangzhou 510632, China; 5 Guangdong Provincial Key Laboratory of Translational Medicine in Lung Cancer, Guangdong Provincial People’s Hospital (Guangdong Academy of Medical Sciences), Southern Medical University, Guangzhou 510080, China; 6 Clinical Research Institute, State Key Laboratory of Complex Severe and Rare Diseases, Peking Union Medical College Hospital, Chinese Academy of Medical Science & Peking Union Medical College, Beijing 100730, China

**Keywords:** Non-small cell lung cancer, MET dysregulation, proteomics, MET inhibitor, biomarker

## Abstract

**Simple Summary:**

Targeted therapy has revolutionized the treatment of non-small cell lung cancer (NSCLC) and MET inhibition is a promising therapy for MET-dysregulated NSCLC. However, due to the lack of effective biomarkers, the clinical efficacy is unsatisfactory. This study aims to investigate the clinical utility of plasma proteomics-derived biomarkers for MET-dysregulated NSCLC (including *MET* amplification and MET overexpression). We analyzed 89 longitudinal plasma samples from MET-dysregulated advanced-stage NSCLC patients treated with MET inhibitors by the method of mass spectrometry. The results showed that the peripheral plasma proteomic characteristics were associated with the outcomes of patients treated with MET inhibitors. Through biomarker selection, we found a four plasma protein signature (MYH9, GNB1, ALOX12B, and HSD17B4 proteins) could predict the response and progression-free survival of patients treated with MET inhibitors with high accuracy. This study highlighted the clinical utilization of plasma biomarkers to scream patients to receive MET inhibitors.

**Abstract:**

MET inhibitors have shown promising efficacy for MET-dysregulated non-small cell lung cancer (NSCLC). However, quite a few patients cannot benefit from it due to the lack of powerful biomarkers. This study aims to explore the potential role of plasma proteomics-derived biomarkers for patients treated with MET inhibitors using mass spectrometry. We analyzed the plasma proteomics from patients with MET dysregulation (including *MET* amplification and MET overexpression) treated with MET inhibitors. Thirty-three MET-dysregulated NSCLC patients with longitudinal 89 plasma samples were included. We classified patients into the PD group and non-PD group based on clinical response. The baseline proteomic profiles of patients in the PD group were distinct from those in the non-PD group. Through protein screening, we found that a four-protein signature (MYH9, GNB1, ALOX12B, HSD17B4) could predict the efficacy of patients treated with MET inhibitors, with an area under the curve (AUC) of 0.93, better than conventional fluorescence in situ hybridization (FISH) or immunohistochemistry (IHC) tests. In addition, combining the four-protein signature with FISH or IHC test could also reach higher predictive performance. Further, the combined signature could predict progression-free survival for MET-dysregulated NSCLC (*p* < 0.001). We also validated the performance of the four-protein signature in another cohort of plasma using an enzyme-linked immunosorbent assay. In conclusion, the four plasma protein signature (MYH9, GNB1, ALOX12B, and HSD17B4 proteins) might play a substitutable or complementary role to conventional MET FISH or IHC tests. This exploration will help select patients who may benefit from MET inhibitors.

## 1. Introduction

The *MET* proto-oncogene has been known to play an important role in promoting tumor cell proliferation, tumor invasion, and metastasis in non-small cell lung cancer (NSCLC) either as a primary oncogenic driver or as a co-driver in the context of acquired resistance to tyrosine kinase inhibitors (TKIs) [1,2,3]. Activation of the MET pathway can be caused by *MET* amplifications, protein overexpression, gene mutations, and fusions [4]. The prevalence of *MET* amplification of NSCLC is 1–5% and 5–20% for *MET de novo* and acquired amplification, respectively [1,5]. MET overexpression is more common in NSCLC, with approximately 20% to 25% of patients identified by immunohistochemistry (IHC) [6,7]. Previous studies have demonstrated that multiple MET inhibitors showed promising efficacy for NSCLC patients with *MET* amplification or MET protein overexpression with an objective response rate of approximately 67% and 68% (IHC3+), respectively [8,9,10]. 

Fluorescence in situ hybridization (FISH) is a standard method to detect *MET* amplification for NSCLC patients. It can distinguish MET focal amplification from *MET* polysomy by calculating both the copies of *MET* per cell and the ratio of *MET* to chromosome (*MET*/CEP7) [11,12]. However, it remains challenging to define an optimal *MET* copy number and *MET*/CEP7 threshold to select eligible patients to receive MET inhibitors. Many FISH-selected patients cannot benefit from MET inhibitors [9,13]. MET overexpression is another potential biomarker for screening patients to be treated with MET inhibitors. Several clinical trials have shown promising efficacy for patients with MET overexpression treated with MET inhibitors plus epidermal growth factor receptor-TKIs (EGFR-TKIs) in the setting of acquired resistance to EGFR-TKIs [8,9]. However, the correlation between MET overexpression and *MET* amplification is poor [14,15]. Thus far, MET overexpression by IHC served as a biomarker for predicting response to MET inhibitor remains controversial. Together, the clinical practice of MET inhibitors is limited by ambiguous diagnostic criteria. Quite a few patients cannot benefit from MET inhibitors owing to the lack of predictive biomarkers with sufficient accuracy to select potentially beneficial patients to receive MET inhibitors. There is an emergent need to find more powerful and easier predictive biomarkers to identify eligible patients who would benefit from MET inhibitors. 

Mass spectrometry (MS)-based proteomics is a high-through and unbiased method for characterizing oncogenic mechanisms and identifying potential prognostic and predictive biomarker [16]. It can detect and quantify tens of thousands of proteins with high specificity, making it an ideal approach for the study of biomarkers identification [17]. A large-scale study investigating the proteogenomics of lung adenocarcinoma revealed the signatures of oncogenesis and successfully identified several novel prognostic and therapeutic biomarker candidates [18]. In addition, MS-based proteomics can also detect plasma proteome by dynamic monitoring, and therefore act as an excellent tool to screen biomarker candidates for multiple diseases. A study used plasma proteomics to identify panels of biomarkers for anti-PD-(L)1 response prediction in NSCLC with an area under the curve (AUC) value of 94.1% [19]. Another study integrating a plasma and paired tissue proteomics approach also identified several noninvasive proteomic biomarkers panels for alcohol-related liver disease with an AUC value of 0.92 [20]. Recent advances in MS-based proteomics technology have greatly extended its application in clinical and translational research [21].

Herein, we conducted a MS-based, data-independent acquisition (DIA) quantitative proteomic approach to explore the blood-based proteomic profiles to determine predictive biomarkers for MET-dysregulated NSCLC patients treated with MET inhibitors. The selected biomarker candidates were further validated by enzyme-linked immunosorbent assay (ELISA) tests in the validation cohort.

## 2. Materials and Methods

### 2.1. Patient Enrollment and Sample Collection

Advanced-stage NSCLC patients with MET dysregulation treated with MET inhibitors were enrolled from 1 October 2014, to 10 April 2019, at Guangdong lung cancer institute. MET dysregulation consisted of MET protein overexpression with MET IHC score ≥270 and *MET* amplification by FISH with mean gene copy number greater or equal to five, and a *MET* to centromere of chromosome 7 (*MET*/CEP7) ratio of 2 or more [12,22]. Tumor response and time to progression were evaluated according to RECIST 1.1. The cut-off date for the last follow-up was 23 June 2020. Samples were collected up to 3 days before MET inhibitors treatment, the best response (about 8–12 weeks) after the initial MET inhibitors treatment, and the disease progression time point. The best response is recorded when patients have the largest tumor shrinkage during treatment, with 30% as partial response and −20–30% as stable disease according to RECIST 1.1 criteria. The best responses often occurred 8–12 weeks after treatment initiation in most of the patients. Progression-free survival (PFS) was defined as the time between the patient receiving treatment in the study and the date of disease progression or censored at the date of the last follow-up according to RECIST 1.1.

Plasma samples were collected in pro-coagulation vacuum tubes using standard venipuncture protocols and were then extracted by centrifugation for 15 min at 2500 rpm. The Plasma samples were stored at −80 °C before use.

### 2.2. Plasma Sample Preparation for Spectral Library Generation

All plasma samples were processed by the Agilent 1290 Infinity II liquid chroma-tography system coupled with the Multi Affinity Removal Column, Human-14 to remove abundant proteins. About 10 µL each sample was taken out and mixed. The mixed sample and all the 89 samples were precipitated by trichloroacetic acid (TCA) solution for about 4 h at 4 °C. After centrifuging at 16,000× *g* for 30 min at 4 °C, the pellets were washed with 500 µL cold acetone three times and dried with a vacuum concentrator (Labconco, Kansas, MO, USA). The dried pellets were dissolved in 40 µL 8 M Urea in 500 mM Tris-HCl buffer (pH 8.5) and ultrasonically treated for 10 min. The samples were reduced with 20 mM (2-carboxyethyl) phosphine hydrochloride (TCEP) (500 mM in 100 mM Tris/HCl pH 8.5) at room temperature for 20 min and alkylated with 40 mM IAA at room temperature in the dark for 30 min. The mixtures were diluted with 200 µL 100 mM Tris-HCl buffer (pH 8.5) followed by adding trypsin at a 1:20 ratio for 16 h. The peptides were desalted and re-dissolved with 50 µL Mil-li-Q water with 0.1 vol% formic acids (FA). The indexed Retention Time (iRT) calibration peptides were spiked into the 89 peptide samples for DIA analysis later. The mixed sample without iRT peptides was separated into two samples, one of which was used for High-PH reversed-phase fraction and quality control (QC) of the DIA analysis later, respectively. The QC sample was added with iRT before analysis.

### 2.3. High-pH Reversed-Phase Fractionation

The mixed peptide sample fractioning was performed on a Chromatographic column (BEH C18, 300A, 1.7 µm, 1 mm × 150 mm) coupled to a Waters XevoTM AC-QUITY UPLC (Waters, Milford, MA, USA) with an 80 min liquid phase gradient. We collected the first 4 min of liquid as the first fraction, the liquid of the 64–68 min as the last fraction, and discarded the liquid of the last 12 min. We collected the liquid sample every minute during the gradient of 4–64 min. The first fraction was mixed with the last one and the rest were mixed in pairs every 30 fractions. Finally, 31 fractions were obtained and vacuum-centrifuge dried. All 31 fractions were reconstituted in 10 µL Milli-Q water with 0.1 vol% formic acids (FA). IRT peptides were spiked before the data-dependent analysis (DDA).

### 2.4. Liquid Chromatography

All the peptide samples were separated on an EASY-nLC1200 liquid chromatography system (ThermoFisher, San Jose, CA, USA) coupled with a 25 cm × 75 μm home-packed analytical column (1.5 μm ReproSil-Pur 120 C18-AQ particles (Dr. Maisch)). Mobile phases A and B were water and 80% ACN with 0.1 vol% formic acids. Samples were analyzed with a 120 min gradient at a flow rate of 300 nL/min and the concentration of B% was increased from 4 to 10% within 4 min, followed by an increase to 30% at 4–103 min and a further increase to 100% at 103–113 min and kept 100% B for the last 7 min.

### 2.5. Mass Spectrometry

All the samples were analyzed on Thermo QExctive HF-X (ThermoFisher, San Jose, CA, USA). The 31 fraction samples obtained through high-Ph reversed-fraction processing were operated in data-dependent mode which was used for the spectral library generation. All 89 plasma samples were analyzed in data-independent mode and the data was used for bioinformatic analysis later. We add a technical QC every 12 samples.

For the DDA runs, the full MS scan was performed with a scan range (m/z) between 300 and 1500 m/z. The MS/MS had a resolution of 60,000. The automatic gain control (AGC) target was 3e6 with a maximum injection time of 50 ms. The HCD dd-MS2 scan selected top 30 intensity peptides and was performed with the following parameters: resolution = 15,000; AGC target = 5e5; maximum injection time = 40 ms, NCE = 30, isolation window = 1.7 m/z.

For the DIA analysis, the full MS-SIM had a resolution of 60,000 and a scan range between 350–1200 m/z. The AGC target was 3e6 and the maximum injection time was set to 50 ms. Each full MS was followed by 64 narrow isolation widths which were named DIA windows. The resolution was set to 30,000 and the AGC target was 1e6.

### 2.6. Generation of Spectral Libraries and DIA Data Analysis

Spectral libraries were generated from the acquired data of the 31 fractions using Spectronaut version 14.0 (Biognosys) with the default parameters. MS/MS spectra were matched against the database which was downloaded from human UNIPROT (only reviewed entries, human 20,421 entries).

### 2.7. Enzyme-Linked Immunosorbent Assay (ELISA)

Human protein ELISA kits were used to detect and quantify plasma levels of specific proteins according to manufacturers’ instructions (SAB signalway ELISA Kit for MYH9 and HSD17B4, and Abebio ELISA Kit for GNB1 and ALOX12B). A total of 100 μL of plasma sample and standard dilutions were added to the precoated plates, and the plates were incubated at 37 °C for 2 h. After three times washing, 100 μL diluted Biotin-Conjugate was added, and the plates were then incubated at 37 ◦C for 1 h. After washing, 100 μL Streptavidin conjugated Horseradish Peroxidase (HRP) was added and incubated at 37 °C for 1 h. 100 μL of Substrate Solution was added and incubated at 37 °C for 10 min. Finally, we added 50 μL of Stop Solution and detected the OD values at 450 nm using microplate spectrophotometer (BIO-RAD, xMark). The determination of OD values from serial dilutions of the standard samples was used to generate a standard curve of each protein and the relative concentrations of samples were calculated. 

### 2.8. Statistical Analysis

Data analysis including data imputation, normalization, and principal component analysis (PCA) was performed in R software (version 4.1.2). The missing value was replaced with a median value. Fold-change of 1.5 and *p*-value of 0.05 were used to filter differentially expressed proteins using the limma package in R. Dot plots of Kyoto Encyclopedia of Genes and Genomes (KEGG) and Gene Ontology (GO) enriched functional pathways were plotted by ClusterProfiler package in R. Significant proteins were used for protein-protein interaction network analysis and network visualization was performed using Cytoscape (version 3.9.1). The Student’s t-test was used to compare the protein levels in the plasma between the two groups. Fisher’s exact test was used to compare two categorical variables. Receiver operator characteristic curves (ROC) analyses were used to assess the overall performance of a test and to compare the performance of two or more other tests. ROC analyses in this study were conducted in pROC package in R using response outcomes and protein intensity values. The AUC value was calculated by the area under the ROC curve and was used to assess the performance of the predictive models. An AUC value of more than 0.8 was considered good. The Youden index, which integrates sensitivity and specificity information, was used to identify the optimal thresholds. The predictive model was constructed using logistic regression in R software. The probability of response was calculated using four protein intensities as the following formula listed.

Logit(p) = log(p/(1 − p)) = −0.087 × MYH9 + 0.497 × GNB1 + 2.015 × ALOX12B − 0.936 × HSD17B4 − 21.520

The predictive *p* value was used to conduct the ROC analysis for the four-protein signature and the corresponding AUC value was calculated. The cut-off value (*p* = 0.68) of the predictive model was calculated by the Youden index. The *p* value of more than 0.68 was considered as the low-risk group in the progression-free survival analyses. 

Survival analysis was performed using Kaplan–Meier survival plot and log-rank test *p*-value were calculated. The hazard ratio was calculated by Cox proportional hazards regression and was used to estimate the ratio of the hazard rate in the two groups. A hazard ratio of 1 indicated that no difference was detected in survival between the two groups. A hazard ratio of greater than one or less than one indicated that survival was worse or better in one of the groups. In the present study, all tests of significance were two-sided, and *p*-value < 0.05 was considered statistically significant. 

## 3. Results

### 3.1. Patient Characteristics and Samples Collection

**Figure 1:**
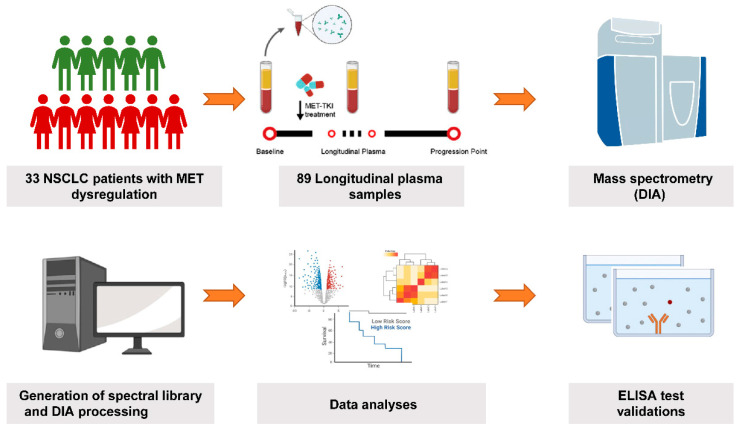
Summary of MET dysregulated NSCLC patients and study workflow.

A total of 33 advanced NSCLC patients diagnosed with MET dysregulation were enrolled in our study including *MET* amplification by FISH test (n = 16) and MET overexpression by IHC test (n = 23). Six patients were positive in both *MET* amplification and MET overexpression. All the patients were treated with MET inhibitors. The clinicopathological characteristics and treatment strategies of the enrolled patients were summarized in Table 1. Of the patients with co-occurrence *EGFR* mutations and MET dysregulation, 39.4% were treated with EGFR-TKIs plus MET inhibitors. No confounders were found between the PD and non-PD groups (Table S1). The disease control rates (DCRs) of patients with *MET* amplification or overexpression were 93.8% and 86.4%, respectively (Table S2). We collected a total of 89 longitudinal peripheral plasma samples at baseline before MET inhibitors treatment (n = 33), best response after treatment (n = 23), and disease progression time point (n = 33, Figure 1). We classified 10 patients who had primary drug resistance to MET inhibitors into the PD group and 23 patients who obtained partial response (PR) or stable disease (SD) into the non-PD group.

### 3.2. Global Proteomic Analysis of Peripheral Plasma and Predictive Biomarkers Selection for Patients Received MET Inhibitors 

**Figure 2:**
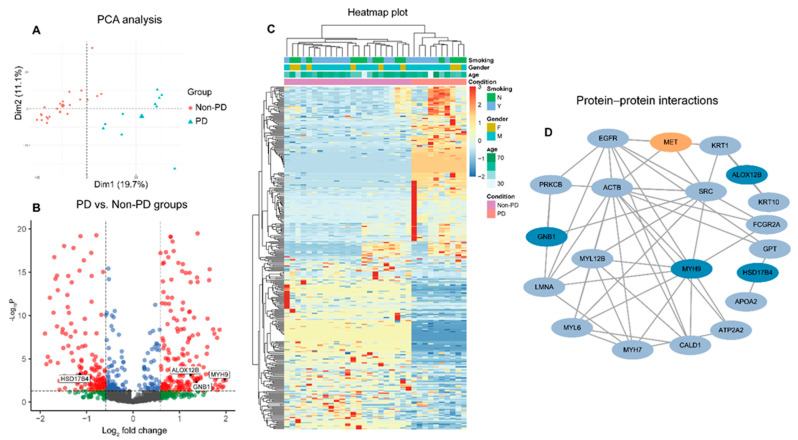
Plasma-based proteomics landscape and biomarkers selection at baseline. (**A**) Principal component analysis of 33 patients; the bigger points in the PD and non-PD groups represent the median value of each group. A volcano plot (**B**) and heatmap plot (**C**) of differentially expressed proteins between PD and non-PD groups; the red points in the volcano plot indicate the proteins that foldchange >1.5 and *p* value < 0.5; the green points indicate the proteins that foldchange >1.5 and *p* value ≥ 0.5; the blue points indicate the proteins that foldchange ≤1.5 and *p* value < 0.5; the grey points indicate the proteins that foldchange ≤ 1.5 and *p* value ≥ 0.5. (**D**) Protein-protein interaction network of differentially expressed proteins.

We performed high-resolution mass spectrometry using a DIA method for the peripheral plasma sample. A total of 1619 proteins were identified from all plasma samples and approximately 1106 unique proteins (range: 914–1296 proteins) were identified in each sample (Figure S1A). The patients in the PD group and non-PD group were clustered independently in unsupervised hierarchical clustering and principal component analysis (PCA), indicating the distinct peripheral proteomic profiles between the two groups at baseline (Figure 2A, Figure S1B). Furthermore, we found a total of 463 differentially expressed proteins and the number of up-regulated proteins was comparable with down-regulated proteins (220 up-regulated vs. 243 down-regulated proteins) (Figure 2B,C). GO and KEGG enrichment analyses revealed that the differentially expressed proteins were enriched in the glycolysis, angiogenesis, Rap1 signaling pathway, cell adhesion, and gap junction, which may contribute to cancer metabolism, migration, and growth (Figure S2). To elucidate the correlation between the differentially expressed proteins and the MET dysregulation pathway, we conducted a protein-to-protein interaction network analysis through the STRING database (Figure 2D). We found a large number of proteins interacted with or regulated by the MET pathway. After manual screening, we found four proteins had greatly higher or lower fold change with significant *p* value in the PD group than the non-PD group (MYH9 = 4.00, *p* = 0.003; GNB1 = 2.53, *p* ≤ 0.003; ALOX12B = 2.40, *p* ≤ 0.001 and HSD17B4 = 0.46, *p* ≤ 0.001).

### 3.3. The Predictive Performance of Biomarkers for Response to MET Inhibitors in MET-Dysregulated NSCLC Patients

**Figure 3:**
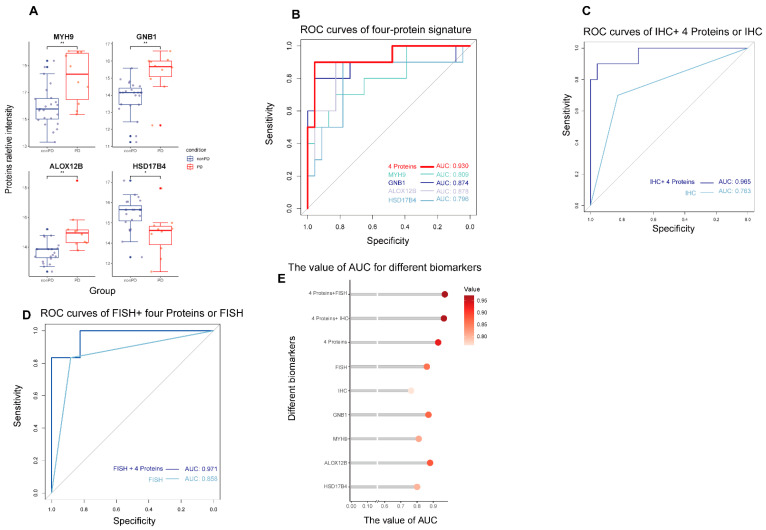
The predictive performance of different models for response to MET inhibitor. (**A**) Boxplots of the relative intensity of four selective protein biomarkers between PD and non-PD groups. * *p* value < 0.05, ** *p* value < 0.01. (**B**) The ROC curves for the performance of four proteins, (**C**) IHC, IHC plus four proteins, (**D**) FISH, and FISH plus four proteins in the prediction of response to MET inhibitors for MET-dysregulated lung cancer patients. (**E**) The AUC values of different models.

We compared the relative protein intensities between the PD and non-PD groups at baseline plasma (Figure 3A). The results showed that MYH9, GNB1, and ALOX12B proteins had significantly higher intensities in the PD group versus those in the non-PD group, representing their potential relation with poor response to MET inhibitors. Another protein, called HSD17B4, was significantly downregulated in the PD group. The predictive performances of the four proteins at baseline were measured by the ROC analysis with AUC values of 0.809, 0.874, 0.878, and 0.796 for the MYH9, GNB1, ALOX12B, and HSD17b4 individual proteins, respectively (Figure 3B). After combining four proteins, the AUC value reached 0.930, which was higher than that of individual proteins and conventional FISH and IHC methods (AUC values: 0.763 and 0.858, respectively; Figure 3C,D). Besides, the addition of four-protein signature to FISH or IHC outperformed the individual FISH or IHC methods, with AUC values of 0.971 and 0.965, respectively (Figure 3C–E).

**Figure 4:**
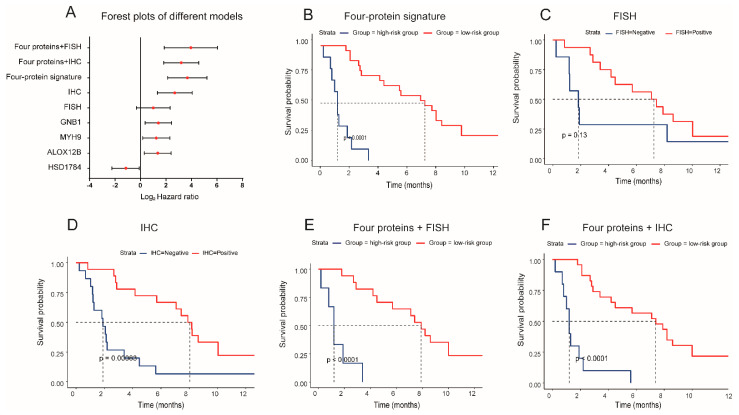
The predictive performance of different models for progression-free survival of patients treated with MET inhibitors. (**A**) Forest plots of hazard ratios and 95% confidence interval in different predictive models. Kaplan–Meier plots of progression-free survival based on the combination of four proteins (**B**), *MET* FISH (**C**), MET IHC (**D**), Four proteins plus FISH (**E**), and four proteins + IHC (**F**).

We further explored the performance of nine proteomic-based models (MYH9, GNB1, ALOX12B, HSD17B4, FISH, IHC, four-proteins signature, four proteins + IHC, four proteins + FISH) in the prediction of PFS in patients who received MET inhibitors (Figure 4A). Based on the ROC analysis and Youden index calculations, the patients were divided into the low-risk group and high-risk group in each of the models. The patients in the low-risk group meant they were more likely to benefit from MET inhibitors and survived longer. The four individual proteins can significantly stratify the PFS of patients treated with MET inhibitors with the hazard ratios (HRs) of MYH9 (HR = 2.35, *p* = 0.024), GNB1 (HR = 2.63, *p* = 0.009), ALOX12B (HR = 2.55, *p* = 0.012), and HSD17B4 (HR = 0.45, *p* = 0.031), respectively. The four-protein signature showed improved predictive performance with an HR of 12.66, 95%CI (4.34, 36.95), P <0.001, better than FISH (HR = 1.99, *p* = 0.13) and IHC (HR = 6.42, *p* = <0.001) methods (Figure 4B–D). The median PFS was 1.2 months for the high-risk group and 7.4 for the low-risk group in the four-protein signature model (Figure 4B). When four proteins were combined with the FISH or IHC test, the models reached higher predictive performances, with HR of 15.39, *p* = <0.001, and HR of 9.1, *p* = <0.001, respectively (Figure 4E,F).

### 3.4. Dynamic Change and Validation of the Four Biomarker Candidates in Plasma following MET Inhibitors Treatment

**Figure 5:**
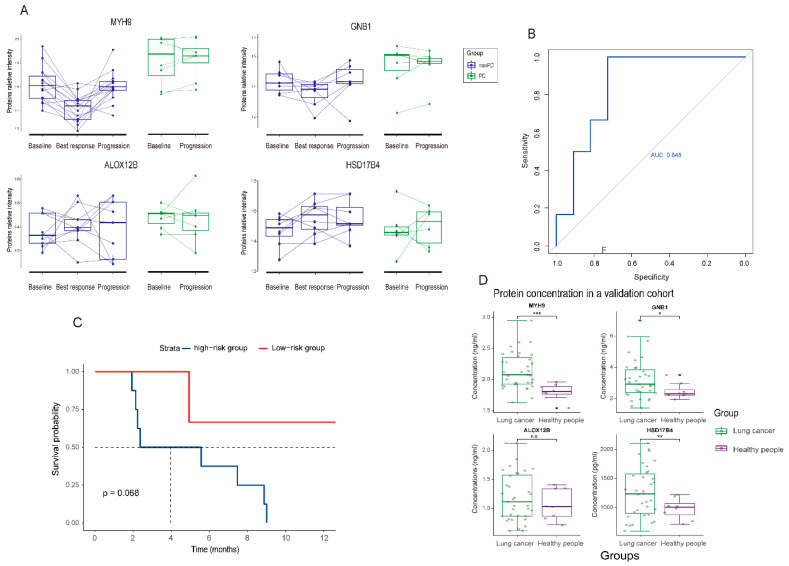
Dynamic change and validation of four biomarker candidates in the prediction of response to MET inhibitors. (**A**) Longitudinal relative proteins intensity at baseline, best response, and progression between non-PD and PD groups. (**B**) Validation of four biomarker candidates in another cohort of MET dysregulated NSCLC patients using plasma ELISA method. (**C**) Kaplan–Meier plots of progression-free survival based on the four-protein signature in the validation group. (**D**) Boxplots of concentrations of the four proteins in lung cancer patients and healthy people. * *p* value < 0.05, ** *p* value < 0.01, *** *p* value < 0.001.

In an attempt to investigate the correlation of four biomarkers with clinical efficacy to MET inhibitors, we also monitored the dynamic change of these four proteins in peripheral plasma (Figure 5A). In non-PD group, three biomarkers (MYH9, GNB1, and ALOX12B) have higher expression levels at baseline; then the intensities dropped at the best response and elevated at the progression. These phenomena indicated that the dynamic changes in the three proteins were negatively associated with the efficacy of MET inhibitors. Regarding the HSD17B4 protein, its intensity was low at baseline, then increased at the best response. In the PD group, the concentrations of the four proteins at baseline and disease progression did not change significantly, indicating the primary resistance to MET inhibitors for these patients. Although we could not exclude the effect of MET inhibitors on the change in protein levels, these phenomena indicated that the dynamic changes of proteins may be largely dependent on the efficacy of MET inhibitors. In addition, the addition of EGFR TKI did not affect the proteomics results (Figure S3). 

We detected the concentration of the four proteins (MYH9, GNB1, ALOX12B, and HSD17B4) in a validation cohort of 17 patients using the ELISA kit. The clinical characteristics of the patients in a validation cohort was described in Table S3. All four proteins can be successfully detected in plasma. Consistent with the results above, the concentrations of MYH9, GNB1 and ALOX12B proteins were higher in the PD group (Figure S4A). However, no statistical significance was observed due to the small sample size. Further, the four-protein signature could predict the response and PFS for patients who received MET inhibitors with an AUC value of 0.848 and HR of 5.82 (*p* = 0.06, Figure 5B,C). The concentrations of the four proteins in lung cancer are significantly higher than those in healthy people, suggesting the change in the four proteins may result in tumor progression (Figure 5D). In 230 patients with adenocarcinoma from the TCGA cohort, higher expressions of MYH9, GNB1, and ALOX12B were associated with poor overall survival outcomes, while higher expression of HSD17B4 was associated with better survival outcomes (Figure S4B) [23]. 

## 4. Discussion

To our knowledge, this is the first study to explore the novel non-invasive predictive biomarkers of the efficacy of MET inhibitors for MET-dysregulated NSCLC patients using MS-based proteomics. We found that the plasma proteomic profiles at baseline were associated with the outcomes of patients treated with MET inhibitors. The combined four-protein signature (MYH9, GNB1, ALOX12B, HSD17B4) in plasma might effectively predict the responses and PFS outcomes of patients who received MET inhibitors, with a high AUC value of 0.930 and an HR of 12.66, *p* <0.001. This study highlights that the four-protein signature might play an alternative or complementary role to MET FISH or IHC method.

Several methods have been examined to select eligible patients for MET inhibitors, including FISH, next-generation sequence (NGS), droplet digital PCR (ddPCR), and IHC methods [11,22,24,25,26]. FISH was currently used to detect *MET* amplification in the clinic, but no consensus regarding the threshold of *MET* signals and *MET*/CEP7 value was defined to date [27]. Besides, the MET signal was distributed variably and the signal clustered or overlapped, making the counting signal difficult [25]. In terms of MET overexpression, the concordance of MET expression and *MET* amplification was low, and its correlation with treatment outcomes remained controversial [14,15]. Both FISH and IHC methods required tissue biopsies, which were not always feasible and put patients at risk. In addition, the heterogeneity of tumor and semi-quantitative FISH and IHC methods are prone to bias and depend on the experience of the pathologist [28]. Other diagnostic methods like NGS and ddPCR cannot distinguish true *MET* amplification from *MET* polysomy and the purity of tumor DNA also affected the results [26,29]. 

To overcome the flaws of traditional diagnostic methods discussed above, we proposed a novel MS-based proteomic method to select the predictive biomarker candidates for patients treated with MET inhibitors. Although DNA biomarkers have been used to guide personalized oncology, most of the small-molecule inhibitors target proteins instead of DNA, such as EGFR-TKIs [30,31]. In this study, we did not screen the biomarkers for patients with *MET* amplification or MET overexpression separately. Instead, we screened the differentially expressed protein biomarkers in plasma that participated in the MET signal pathway which were also associated with the treatment outcome. Previous studies have demonstrated that a subset of proteins in plasma can be secreted from or interact with the primary tumor [32,33,34]. Consistent with previous results, the proteomics profiles in our study were distinct between the patients in the PD group and those in the non-PD group. Further, through thousands of protein screenings, we identified four proteins (MYH9, GNB1, ALOX12B, HSD17B4) that can predict the response to MET inhibitors for MET-dysregulated lung cancer patients. The four-protein signature showed a higher predictive performance than the FISH or IHC methods, with AUC values of 0.930 vs. 0.858 or 0.763. The FISH-positive and IHC-positive patients showed the DCRs of 93.8% and 86.4%, which were consistent with results in the INSIGHT trial [9]. The positive group in our four-protein signature demonstrated a higher DCR of 95.7%, indicating the higher predictive performance of our model. The model also outperformed FISH and IHC methods in the prediction of PFS for patients treated with MET inhibitors, with a hazard ratio of 12.66 vs. 1.99 or 6.42. The addition of the four-protein signature to FISH or IHC methods could reach higher predictive performance, from the AUC values of 0.763 and 0.858 to 0.971 and 0.965, respectively. Therefore, the four-protein signature in our study not only represented an independent biomarker, but also a complementary biomarker to the FISH and IHC methods.

We integrated the downstream proteins as a predictive signature, as the single protein dysregulation may not fully represent the abnormality of a pathway. The biological functions of the four proteins have been reported to be associated with cancer development and progression. Previous studies showed that the MYH9 protein could act as a promoter of tumor stemness that facilitates tumor pathogenesis through the regulation of Wnt-β-catenin-STAT3 signaling, which can further interact with the MET pathway [35]. High expression of MYH9 conferred a poor prognosis for hepatocellular carcinoma, which was consistent with our results [36]. In our study, MYH9 enriched in angiogenesis and cell-cell junction, indicating its role in tumor progression. GNB1 protein played an important role in the PI3K/mTOR-related anti-apoptosis pathway, conferring transformed and resistance phenotypes across a range of human tumors. Acquiring mutations in the *GNB1* gene could cause resistance to tyrosine kinase inhibitors for leukemia [37]. ALOX12B was involved in lipid deoxygenation and the breakdown of amino acids. It promoted cell proliferation via regulation of the PI3K/ERK1 signaling pathway and was associated with survival outcomes in cancers [38,39]. HSD17B4 is a molecule involved in the peroxisome pathway and epithelial cell development [40]. Previous studies showed that HSD17B4 was highly expressed in most human cancers and was significantly associated with treatment efficacy [41,42]. Together, the four proteins are downstream molecules of the MET pathway, indicating the biological connection to the MET signaling.

To confirm the predictive performance of the four-protein signature, we monitored the dynamic change of circulated plasma-based proteomics, especially focusing on the four biomarker candidates. The results showed that protein intensities were associated with the efficacy of the patient treated with MET inhibitors. MYH9, GNB1, and ALOX12B were negatively associated with the tumor response, which represented biomarkers of poor outcomes, while HSD17B4 was positively associated with the tumor response. We also used the ELISA test to validate the concentration of four proteins in a validation cohort. The results showed a similar tendency with an AUC value of 0.848. The model also showed an encouraging performance in the prediction of PFS despite the small sample size. In addition, the results from ELISA also demonstrated the clinical utilities of the four-protein signature as a convenient, non-invasive tool to screen eligible patients for MET inhibitors as ELISA was readily available in most molecular laboratories. Overall, this study can select those patients that did not benefit from MET inhibitors and give them other treatments (such as chemotherapy, angiogenesis inhibitors, or immunotherapy), which can improve their survival outcomes.

Among the limitation of this study, firstly the sample sizes are relatively small with only 33 patients enrolled in our study, which may cause an overfit in our predictive models. Some results in the validation cohort were not significant may also attribute to the small sample size. It is hard to enroll large-scale patients with thorough clinical characteristics, serial plasma sample collection, and longtime follow-up. However, the dynamic change of the four proteins and the ELISA results can consolidate our findings. Secondly, the sample collections had heterogeneity, with a long period of collection time from 2014 to 2019, which may cause discrepant results in our study. Thirdly, the relationship of the four proteins with primary lung cancer tissue remained unknown. Due to the limited tumor tissue, we cannot perform the IHC test for primary cancer tissue to verify the origins of the four proteins.

## 5. Conclusions

The peripheral plasma proteomic characteristics were associated with the outcomes of MET-dysregulated patients treated with MET inhibitors. A combination of plasma MYH9, GNB1, ALOX12B, and HSD17B4 proteins could effectively and robustly predict the responses and PFS of patients receiving MET inhibitors, with a substitutable or complementary role to conventional MET FISH or IHC tests. This exploration will help select patients who may benefit from MET inhibitors.

## Figures and Tables

**Table 1:** The clinicopathological characteristics and treatment strategies of the enrolled patients.

Clinical characteristics		Overall
		(n = 33)
Age		
	Median [Range]	58.4 [29.3–73.5]
Gender (%)		
	Female	8 (24.2%)
	Male	25 (75.8%)
Smoking history (%)		
	No	14 (42.4%)
	Yes	19 (57.6%)
Pathology (%)		
	Adenocarcinoma	32 (97.0%)
	Pulmonary sarcomatoid carcinoma	1 (3.0%)
Stage (%)		
	III	1 (3.0%)
	IV	32 (97.0%)
Performance status score (%)		
	1	32 (97.0%)
	2	1 (3.0%)
Brain metastasis (%)		
	No	23 (69.7%)
	Yes	10 (30.3%)
*EGFR* mutation (%)		
	19DEL	5 (15.2%)
	L858R	8 (24.2%)
	Negative	20 (60.6%)
*MET* FISH (%)		
	Negative	7 (21.2%)
	Positive	16 (48.5%)
	NA	10 (30.3%)
MET IHC (%)		
	Negative	11 (33.3%)
	Positive	22 (66.7%)
Treatment (%)		
	MET inhibitor + EGFR-TKI	12 (36.4%)
	MET inhibitor	21 (63.6%)
Treatment line (%)		
	1	7 (21.2%)
	≥2	26 (78.2%)

## Data Availability

Public database with RNA sequencing data could be obtained online. The proteomics data were uploaded in the supplementary material.

## Supplementary Materials

The following supporting information can be downloaded at: https://www.mdpi.com/article/10.3390/cancers15010302/s1, Figure S1: (A) Bar plot for the detected numbers of each sample at baseline. (B) Hierarchical cluster of the samples at baseline. Figure S2: GO and KEGG enrichment analyses of differentially expressed proteins between the PD and non-PD groups. Figure S3: The relative intensities of four selective protein biomarkers between patients treated with MET inhibitor with or without EGFR-TKI. Figure S4: (A) The concentrations of four proteins in the validation group using the ELISA method. (B) The prognostic value of four proteins for overall survival in the TCGA dataset. Table S1: The clinicopathological characteristics and treatment strategies of the enrolled patients based on the PD and non-PD groups. Table S2: Disease control rates and objective response rates in groups stratified by different models. Table S3: The clinicopathological characteristics and treatment strategies of the enrolled patients in the validation cohort.
