# Supplementary material for: Longitudinal Plasma Proteomics-Derived Biomarkers Predict Response to MET Inhibitors for MET-Dysregulated NSCLC
Source: Cancers (Basel). 2023 Jan 1;15(1):302. doi: 10.3390/cancers15010302 (PMC9818927; doi:10.3390/cancers15010302)
Supplement: Supplementary file 1 [file cancers-15-00302-s001.zip › supplementary materials.pdf]

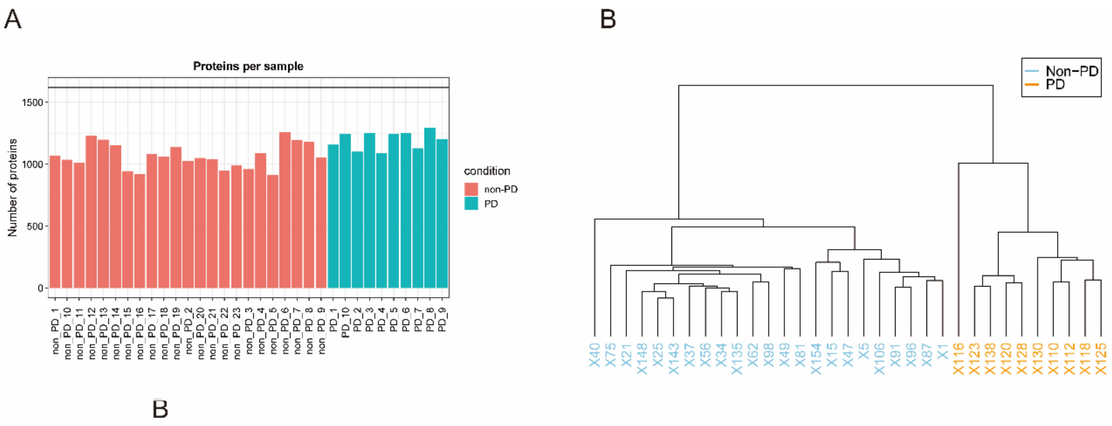

Figure S1: (A) Bar plot for the detected numbers of each sample at baseline. (B) Hierarchical cluster of the samples at baseline.

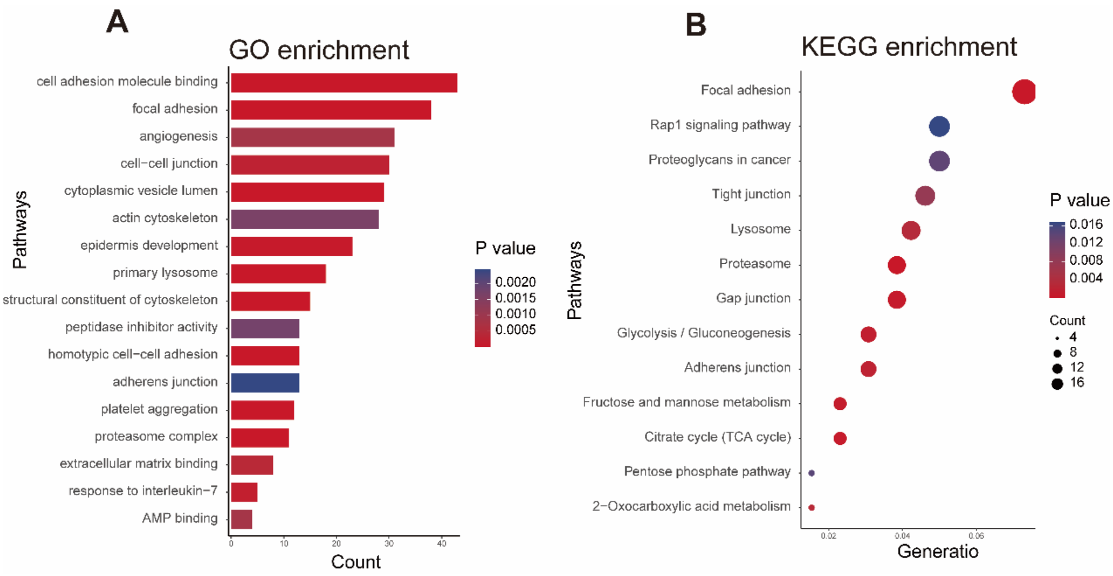

Figure S2: GO and KEGG enrichment analyses of differentially expressed proteins between the PD and non-PD groups.

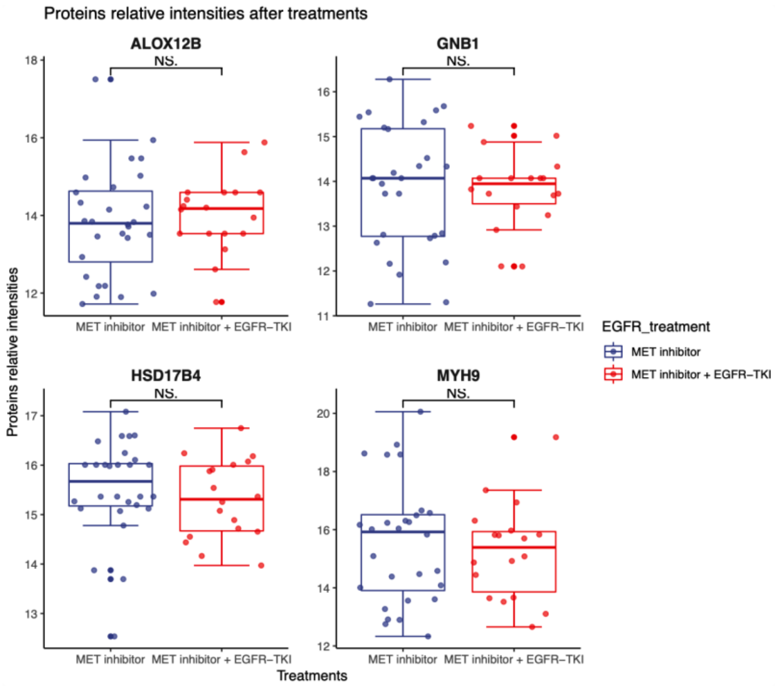

Figure S3. The relative intensities of four protein biomarkers between patients treated with MET inhibitor with or without EGFR-TKI.

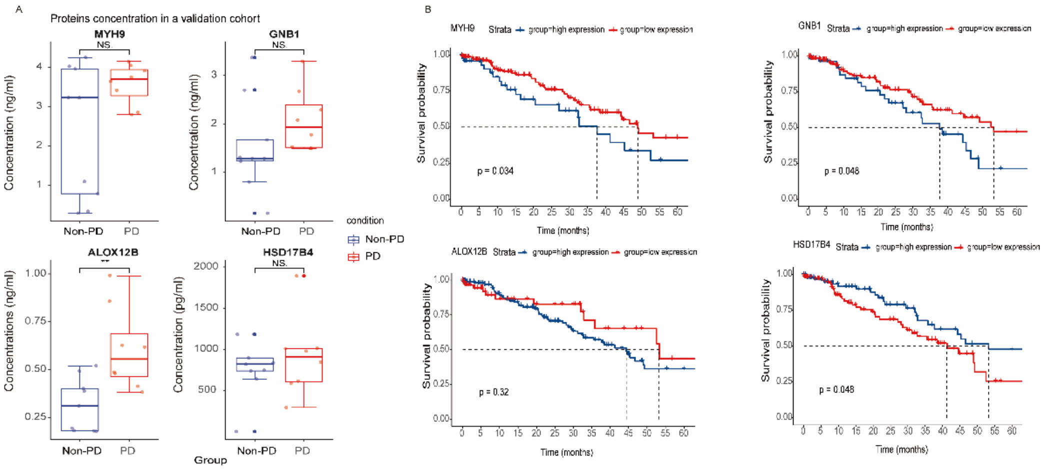

Figure S4: (A) The concentrations of four proteins in the validation group using ELISA method. (B) The prognostic values of four proteins for overall survival in TCGA dataset.

Table S1. The clinicopathological characteristics and treatment strategies of the enrolled patients based on the PD and non-PD groups.

Clinical characteristics	Non-PD group n=23 (%)	PD group n=10 (%)	P value
Age			0.829
Median [Range]	57.9 (31.9, 67.9)	56.8 (29.3, 73.5)	
Gender (%)			1
Female	6 (26.1)	2 (20.0)	
Male	17 (73.9)	8 (80.0)	
Smoking history(%)			1
No	10 (43.5)	4 (40.0)	
Yes	13 (56.5)	6 (60.0)	
Pathology (%)			1
Adenocarcinoma	22 (95.7)	10 (100.0)	
Pulmonary sarcomatoid carcinoma	1 (4.3)	0 (0.0)	
Stage (%)			1
III	1 (4.3)	0 (0.0)	
IV	22 (95.7)	10 (100.0)	
Performance status score (%)			1
1	22 (95.7)	10 (100.0)	
2	1 (4.3)	0 (0.0)	
Brain metastasis (%)			1
No	16 (69.6)	7 (70.0)	
Yes	7 (30.4)	3 (30.0)	
EGFR mutation (%)			0.1
19DEL	5 (21.7)	0	
L858R	7 (30.4)	1 (10.0)	
Negative	11 (47.8)	9 (90.0)	
MET FISH (%)			0.035
Negative	2 (8.7)	5 (50)	
Positive	15 (65.2)	1 (10)	
NA	6 (26.1)	4 (40.0)	
MET IHC (%)			0.006
Negative	4 (17.4)	7 (70.0)	
Positive	19 (82.6)	3 (30.0)	
Treatment (%)			0.054
MET inhibitor + EGFR-TKI	11 (47.8)	1 (10.0)	
MET inhibitor	12 (52.2)	9 (90.0)	
Treatment line (%)			
1	6 (26.1)	1 (10.0)	0.3968
⩾2	17 (73.9)	9 (90.0)	

NA, not applicable

Table S2: Disease control rates and objective response rates in groups stratified by different models.

	Disease control rate		Objective response rate	
	Positive/Low-risk group	Negative/High risk group	Positive/Low-risk group	Negative/High risk group
FISH	93.8%	28.6%	68.8%	28.6%
IHC	86.4%	36.4%	69.6%	9.1%
Four-protein signature	95.7%	10.0%	69.6%	10.0%
FISH + Four-protein signature	94.4%	0.0%	72.2%	0.0%
IHC + Four-protein signature	95.7%	10.0%	73.9%	0.0%

Table S3: The clinicopathologic characteristics and treatment strategies of the enrolled patients in the validation cohort.

	Non-PD	PD	
**Clinical characteristics**	n=11 (%)	n=6 (%)	P value
Age			0.96
Median [Range]	57.00 [46, 76]	61.00 [48, 78]	
Gender (%)			0.102
Female	5 (45.5)	0 (0.0)	
Male	6 (54.5)	6 (100.0)	
Smoking history (%)			0.6
No	8 (72.7)	3 (50.0)	
Yes	3 (27.3)	3 (50.0)	
Pathology (%)			1
Adenocarcinoma	10 (90.9)	6 (100.0)	
Adenosquamous carcinoma	1 (9.1)	0 (0.0)	
Stage (%)			-
IV	11 (100.0)	6 (100.0)	
Performance status score (%)			0.676
1	8 (72.7)	6 (100.0)	
2	2 (18.2)	0 (0.0)	
3	1 (9.1)	0 (0.0)	
Brain metastasis (%)			0.304
No	5 (45.5)	5 (83.3)	
Yes	6 (54.5)	1 (16.7)	
EGFR mutation (%)			0.43
19DEL	5 (45.5)	3 (50.0)	
L858R	6 (54.5)	2 (33.3)	
Negative	0 (0.0)	1 (16.7)	
MET FISH (%)			0.076
Negative	5 (45.5)	0 (0.0)	
Positive	3 (27.3)	1 (16.7)	
NA	3 (27.3)	5 (83.3)	
MET IHC (%)			0.017
Negative	6 (54.5)	3 (50.0)	
Positive	5 (45.5)	0 (0.0)	
NA	0 (0.0)	3 (50.0)	
Treatment (%)			0.28
MET inhibitor + EGFR-TKI	2 (18.2)	3 (50.0)	
MET inhibitor	9 (81.8)	3 (50.0)	
Treatment line (%)			1
1	3 (27.3)	1 (16.7)	
⩾2	8 (72.7)	5 (83.3)	

NA: not applicable
